# Regulatory B Cells (Bregs) Inhibit Osteoclastogenesis and Play a Potential Role in Ameliorating Ovariectomy-Induced Bone Loss

**DOI:** 10.3389/fimmu.2021.691081

**Published:** 2021-06-30

**Authors:** Leena Sapra, Asha Bhardwaj, Pradyumna Kumar Mishra, Bhavuk Garg, Bhupendra Verma, Gyan C. Mishra, Rupesh K. Srivastava

**Affiliations:** ^1^ Department of Biotechnology, All India Institute of Medical Sciences (AIIMS), New Delhi, India; ^2^ Department of Molecular Biology, ICMR-National Institute for Research in Environmental Health, Bhopal, India; ^3^ Department of Orthopaedics, All India Institute of Medical Sciences (AIIMS), New Delhi, India; ^4^ National Centre for Cell Science (NCCS), Pune, India

**Keywords:** regulatory B cells, Bregs, osteoclast, osteoporosis, bone health

## Abstract

Increasing evidence in recent years has suggested that regulatory B cells (Bregs) are one of the crucial modulators in various inflammatory disease conditions. However, no study to date has investigated the significance of Bregs in modulating osteoclastogenesis. To the best of our knowledge, in the present study, we for the first time examined the anti-osteoclastogenic potential of Bregs under *in vitro* conditions and observed that Bregs suppress RANKL-induced osteoclastogenesis in a dose-dependent manner. We further elucidated the mechanism behind the observed suppression of osteoclasts differentiation *via* Bregs. Our results clearly suggested that the observed anti-osteoclastogenic property of Bregs is mediated *via* the production of IL-10 cytokine. Next, we explored whether Bregs have any role in mediating inflammatory bone loss under post-menopausal osteoporotic conditions in ovx mice. Remarkably, our *in vivo* data clearly suggest that the frequencies of both CD19^+^IL-10^+^ Bregs and CD19^+^CD1d^hi^CD5^+^IL-10^+^ “B10” Bregs were significantly reduced in case of osteoporotic mice model. Moreover, we also found a significant reduction in serum IL-10 cytokine levels in osteoporotic mice, thereby further supporting our observations. Taken together, the present study for the first time establishes the direct role of regulatory B cells in modulating osteoclastogenesis *in vitro*. Further, our *in vivo* data suggest that modulations in the percentage of Bregs population along with its reduced potential to produce IL-10 might further exacerbate the observed bone loss in ovx mice.

## Introduction

B cells are classically characterized by their potential to produce and secrete antibodies. They also function as antigen-presenting cells (APCs) and secrete several immunomodulatory cytokines. Nevertheless, B cells with immunosuppressive functions, called Regulatory B cells or “Bregs”, have also been reported. It is known that the microenvironment plays a crucial role in directing the development and differentiation of Bregs (1, 2). It has also been observed that antigen and B cell receptor (BCR) signaling are crucial for the development of Bregs. In the presence of toll-like receptor (TLR) ligands, such as lipopolysaccharide (LPS), CpG, and CD40L, the development of Bregs can be optimized (3, 4). These Bregs exhibit the ability to regulate various disease conditions including inflammatory bone loss diseases such as rheumatoid arthritis (RA), collagen-induced arthritis (CIA), etc. (5, 6). Bregs *via* the production of interleukin (IL)-10, IL-35, and transforming growth factor (TGF)-β suppress several immunopathologies by barring the expansion of various immune cells, including T lymphocytes (7). Given that IL-10 producing B cells play a crucial role in immune regulation, Tedder et al. defined a subset of Bregs named “B10 cells” whose anti-inflammatory potential is only attributable to the production of IL-10 cytokine in various disease models such as cancer, autoimmune diseases, and infectious diseases. The co-expression of CD1d and CD5 has later been utilized to characterize splenic B10 cells to inhibit the progression of inflammation upon stimulation for 5h with LPS, phorbol 12-myristate 13-acetate (PMA), ionomycin, and monensin under *ex vivo* conditions (8, 9). Various studies demonstrated that the adoptive transfer of splenic B10 cells dampened the autoimmune reactions in several models of experimental autoimmune encephalitis (EAE), CIA, intestinal inflammation, and systemic lupus erythematosus (SLE) (5, 10–12). Thus, multiple studies in both humans and mice highlighted that Bregs suppress inflammatory reactions *via* IL-10 cytokine.

Osteoporosis is a systemic skeletal disease that is mainly characterized by deterioration of bone tissue and low bone mass. Various factors act synergistically to enhance the likelihood of developing osteoporosis viz. age, sex, hormonal imbalance, dietary factors, and the immune system. Accumulating evidence has supported the theory that bone destruction observed in osteoporosis caused by osteoclasts is due to impairment or loss of homeostatic balance of inflammatory and anti-inflammatory cells viz. Th17 and Tregs (13, 14). Various studies proposed that CD4^+^Foxp3^+^ Treg cells *via* secretion of IL-10 cytokine suppress osteoclastogenesis and thus bone resorption (13–16). It has also been reported that numerical defect in the Tregs population along with its efficacy to produce IL-10 cytokine results in the development of various inflammatory bone loss conditions, including osteoporosis. IL-10 is thus not just a signature cytokine for Bregs but also a potent modulator of the immune response that further contributes to the maintenance of bone health *via* inhibition of osteoclast-mediated bone resorption (17). Interestingly, a study reported that Breg cells mediate their immunosuppressive functions by inducing differentiation of naïve T cells into Tregs (18). Nevertheless, the role of Bregs and their secretory cytokines on the differentiation of osteoclasts has not been investigated to date. With the growing involvement of the immune system in the pathology of osteoporosis, our group has recently coined the term “Immunoporosis” that emphasizes the specific role of immune cells in osteoporosis (19).

In the present study, we report that under *in vitro* conditions, IL-10 producing Bregs inhibit the differentiation of osteoclasts. In addition, we also report that the frequencies of Bregs (CD19^+^IL-10^+^ and CD19^+^CD1d^hi^CD5^+^IL-10^+^ “B10 cells”) are significantly reduced in both bone marrow and spleen in ovariectomy-induced post-menopausal osteoporotic mice model. Altogether our results suggest that numerical defects in Bregs along with its inability to secrete IL-10 cytokine might be a contributing factor towards the establishment of pro-inflammatory conditions in the case of postmenopausal osteoporosis. In summary, our results for the first time explore the “Immunoporotic” role of Bregs in bone health.

## Materials and Methods

### Reagents and Antibodies

The following antibodies/kits were obtained from eBiosciences (USA): PerCp-Cy5.5 Anti-Mouse-CD19 (1D3) (45-0193-82), PE-Cy7 Anti-Mouse-CD5 (53-7.3) (25-0051-81), APC Anti-Mouse-CD1d (1B1) (17-0011-82), Foxp3/Transcription factor staining buffer (0-5523-00), and RBC lysis buffer (00-4300-54). FITC Anti-Mouse-IL-10 (JES5-16E3) (505005) was purchased from Biolegend (USA). *InVivo*MAb anti-mouse IL-10 antibody (BE0049) and *InVivo*MAb Rat IgG1 isotype control were purchased from BioXcell (USA). Protein transport inhibitor cocktail and the Mouse TNF-α (560478) ELISA kit were procured from BD (USA). The following ELISA kits were brought from R&D and Elabscience: Mouse IL-10 (M1000B) (E-EL-M0046) and Mouse IL-17 (M1700). PMA, Ionomycin, LPS (*Escherichia coli* serotype 0111: B4), FITC-Phalloidin (P5282), DAPI, and Acid phosphatase leukocyte (TRAP) kit (387A) were procured from Sigma-Aldrich (USA). Macrophage-colony stimulating factor (M-CSF) (300-25) and Receptor activator of nuclear factor κB-ligand (sRANKL) (310-01) were procured from PeproTech (USA). α-Minimal essential media (MEM) and RPMI-1640 were obtained from Gibco (Thermo Fisher Scientific, USA).

### B Cell Purification and Activation

Splenic B cells from C57BL/6 mice were purified by magnetic separation according to the manufacturer’s instructions. Briefly, after RBC lysis, the resulting cells were subjected to a biotinylated mouse B cell enrichment cocktail (BD, USA) and incubated for 20-30 min at 4°C. Labeled cells were then washed carefully with washing buffer and incubated with streptavidin particles plus-DM for 30 min at 4°C. Further, cells underwent magnetic separation using a cell separation magnet, and a negative fraction comprised of resulting B cells was assessed for purity (>95%) by flow cytometric analysis. Purified B cells (>95%) were then cultured in 24-well plates (2 X 10^6^) in 1ml/well in the presence and absence of LPS (10 ug/ml) for different time periods (5h and 24h) at 37°C in 5% humidified CO_2_ incubator. At the end of incubation, LPS induced Bregs were harvested and washed thrice with 1X PBS containing 2% fetal bovine serum (FBS), especially to remove the traces of LPS before co-culturing with bone marrow cells (BMCs) for osteoclasts differentiation.

### Co-Culture of Bregs With BMCs for Osteoclastogenesis

Generation of osteoclasts from mouse BMCs was performed as previously described (16). Briefly, BMCs were harvested from femur and tibiae of 8–10 wks old mice and RBC lysis was performed with 1X RBC lysis buffer. After RBC lysis, cells were cultured overnight in T-25 flask in endotoxin-free α-MEM media supplemented with 10% heat-inactivated FBS and M-CSF (35 ng/ml). On the following day, non-adherent cells (BMCs) (1 X 10^6^ cells/well) were collected and co-cultured with either Bregs or non-activated B cells (1X10^5^-1X10^6^ cells/well) in 24 well plate in different ratios (10:1, 5:1 and 1:1) in the presence of M-CSF (30 ng/ml) and RANKL (100 ng/ml) for 4 days. At an interval of 2 days, media was replenished with fresh media supplemented with M-CSF and RANKL factors. After 4 days of incubation, tartrate-resistant acid phosphatase (TRAP) staining was performed.

For the transwell experimental setup, transwell chambers with 8 µM pore size membranes were employed to physically separate Bregs or B cells from BMCs. Bregs or B cells were seeded in the upper chamber and BMCs in the lower chamber at different cell ratios. After 4 days of incubation, cells were processed for evaluating osteoclastogenesis *via* TRAP staining.

For the IL-10 neutralization experimental setup, neutralizing monoclonal antibody against IL-10 (10 ug/ml) was added in the cocultures of BMCs and Bregs at a 1:1 ratio in the presence of M-CSF (30 ng/ml) and RANKL (100 ng/ml). Isotype control for anti-IL-10 was also added in the control group. After 4 days of incubation, cells were processed for evaluating osteoclastogenesis *via* either TRAP staining or F-actin ring formation assay (described later).

### Tartrate Resistant Acid Phosphatase (TRAP) Staining

For evaluating the generation of mature multinucleated osteoclasts TRAP staining was performed according to the manufacturer’s instructions. Briefly, at the end of incubation cells were washed thrice with 1X PBS and fixed with a fixative solution comprised of citrate, acetone, and 3.7% formaldehyde for 10 min at 37°C. After washing twice with 1X PBS, fixed cells were stained for TRAP at 37°C in dark for 5–15 min. Multinucleated TRAP-positive cells with ≥ 3 nuclei were considered as mature osteoclasts. TRAP-positive multinucleated cells were further counted and imaged using an inverted microscope (ECLIPSE, TS100, Nikon). The area of TRAP-positive cells was quantified with the help of Image J software (NIH, USA).

### F-Actin Ring Formation Assay

F-actin ring formation assay was performed as described previously (16). Briefly, Bregs and BMCs were co-cultured on glass coverslips in a 12-well plate, and after 4 days of incubation, cells were processed for F-actin ring staining. At the end of incubation, after washing cells twice with 1X PBS, cells were fixed with 4% paraformaldehyde (PFA) for 20 min and permeabilized with 0.1% triton X-100 for 5 min. Furthermore, to block non-specific binding, cells were blocked with 1% BSA for 30 min. After the blocking step, cells were stained with FITC-labelled-phalloidin for 1h at room temperature in dark. Finally, after washing nuclei were stained with DAPI (10 ug/ml) and incubated for 5 min in the dark. Subsequently, after mounting slides were observed under an immunofluorescence microscope (Imager.Z2, Zeiss) for F-actin ring formation at 10X magnification.

### Flow Cytometric Analysis

Cells were harvested and stained with antibodies specific to Bregs. For analysis of intracellular IL-10 cytokine by B cells, isolated splenocytes or purified B cells were resuspended in RPMI-1640 complete media comprised of 10% FBS and 0.1% mercaptoethanol (ME) at a density of 2 X 10^6^ cells/ml in 24-well plates. Cells were then activated with LPS (10 ug/ml), PMA (50 ng/ml, Sigma Aldrich), Ionomycin (500 ng/ml, Sigma Aldrich), and a protein transport inhibitor cocktail (BD, USA) for 5h. For surface marker staining, cells were first incubated with anti-CD19-PerCP-Cy5.5, anti-CD5-PE-Cy7, and anti-CD1d-APC and incubated for 30 min in dark on ice. After washing, cells were fixed and permeabilized with 1X fixation-permeabilization-buffer for 30 min on ice in dark. Further, cells were stained with anti-IL-10-FITC for 45 min. After washing, cells were acquired on either BD FACS-AriaIII or BD-LSRFortessa (USA). Flowjo-10 (TreeStar, USA) software was used to analyze the samples, and gating strategy was done as per previously defined protocols.

### Post-Menopausal Osteoporotic Mice Model

All *in vivo* experiments were carried out in 8-10 wks old female C57BL/6 mice. All the mice were housed under specific pathogen-free conditions at the animal facility of All India Institute of Medical Sciences (AIIMS), New-Delhi-India. Following groups were taken for the present study viz. sham (control) and ovariectomized (ovx) (n=6/grp). A healthy control group (sham) was subjected to sham surgery. In the ovx group, mice were exposed to bilateral-ovariectomy after anesthetizing them with ketamine (100-150 mg/kg) and xylazine (5-16 mg/kg) intraperitoneally. Both the groups were maintained on a 12-h light/dark cycle in polycarbonate cages and fed with sterilized food and autoclaved water *ad-libitum*. At the end of the experiment (6 wks), mice were euthanized by carbon dioxide asphyxiation, and blood, bones, and lymphoid tissues were harvested for further analysis. All the procedures were performed in accordance with the principles, the recommendation, and after the due approval of protocol submitted to the Institutional Animal Ethics Committee of AIIMS, New Delhi, India (85/IAEC-1/2018 and 196/IAEC-1/2019).

### Scanning Electron Microscopy (SEM)

SEM was performed for scanning the surface of the femur cortical region of bones (13). Briefly, for 2-3 days bone samples were stored in 1%-Triton-X-100, and, later on, bones were transferred in 1X PBS buffer till the analysis was carried out. After the preparation of bone slices, samples were dried and sputter coating was performed. Subsequently, bones were scanned and imaged using Quanta 200 FEG SEM. SEM images were digitally photographed at 50,000 X magnification to capture the best cortical region of bones. The SEM images were further analyzed through MATLAB software (Mathworks, Natick, MA, USA).

### Micro-Computed Tomography (µ-CT) and Bone Mineral Density (BMD) Measurements

µ-CT scanning and BMD analysis were performed as described previously (13, 14). Briefly, after placing all the samples at the correct orientation, scanning was carried out at 50 kV, 201 mA using a 0.5 mm aluminum filter, and exposure was set to 590 ms. For reconstruction of images NRecon software was used. For trabecular region analysis, ROI was drawn at a total of 100 slices in secondary spongiosa at 1.5 mm from the distal border of growth plates excluding the parts of cortical bone and primary spongiosa. For measuring and calculating the microarchitectural parameters of bone samples CTAn software was employed. Several 3D-histomorphometric indices were obtained such as bone volume/tissue volume ratio (BV/TV); trabecular thickness (Tb. Th); trabecular number (Tb. No.); connectivity density (Conn. Den); trabecular separation (Tb. Sep.); trabecular pattern factor (Tb. Pf.); total cross-sectional area (Tt. Ar.); total cross-sectional perimeter (T. Pm); cortical bone area (Ct. Ar); bone perimeter (B. Pm); and average cortical thickness (Ct. Th). The volume of interest (VOI) of µ-CT scans was used to calculate the BMD of lumbar vertebrae-5 (LV-5), femoral, and tibial bones. BMD was measured by using hydroxyapatite phantom rods of 4 mm diameter with known BMD (0.25 g/cm^3^ and 0.75 g/cm^3^) as a calibrator.

### Enzyme-Linked Immunosorbent Assay (ELISA)

ELISA was carried out for quantitative estimation of the following cytokines viz. IL-10, IL-17, and TNF-α in blood serum using commercially available kits as per the manufacturer’s instructions. Secretion of IL-10 cytokine was also evaluated in the culture supernatants of LPS induced purified B cells and co-cultures of Bregs and BMCs under various conditions. Supernatants were collected and stored at -80^0^C until being measured by ELISA.

### Statistical Analysis

Statistical differences between sham and ovx mice groups were assessed by using analysis of variance (ANOVA) with succeeding comparisons *via* student t-test paired or unpaired as appropriate. We performed an analysis of significance in Sigma Plot (Systat Software, Inc., Germany). All the data values are articulated as Mean ± SEM (n=6). Statistical significance was determined as p ≤ 0.05 (*p < 0.05, **p < 0.01, ***p < 0.001) with respect to the indicated group.

## Results

### Induction of IL-10 Producing Bregs

**Figure 1:**
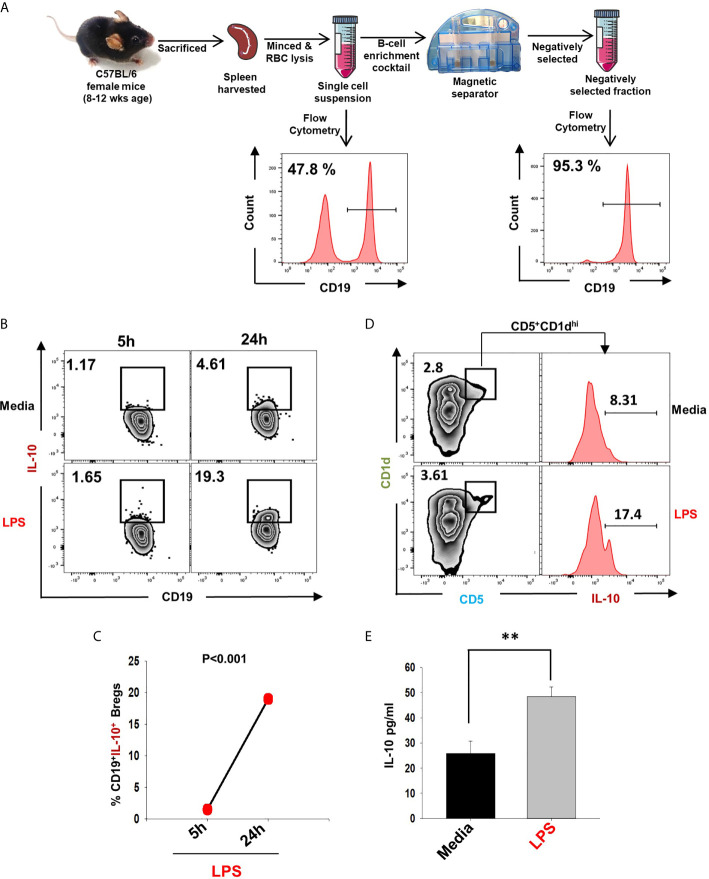
Induction of IL-10 producing Bregs. **(A)** Spleen was harvested and processed for negative selection of CD19^+^ B cells. After estimation of B cells purity (>95 %), cells were stimulated with LPS (10 ug/ml) for different time periods (5h and 24h). **(B)** Zebra plot depicting the percentages of CD19^+^IL-10^+^ in media and LPS after 5h and 24h of stimulation. **(C)** Line plot showing CD19^+^IL-10^+^ B cells after 5h and 24h of LPS stimulation. **(D)** Zebra plot depicting the percentages of CD19^+^CD5^+^CD1d^hi^ B10 cells and histograms depicting IL-10 production by these cells. **(E)** ELISA results of culture supernatants showing levels of IL-10 cytokine. The above images are indicative of one experiment and similar results were obtained in three independent experiments. The results were evaluated by ANOVA with subsequent comparisons by Student t-test for paired or nonpaired data. Statistical significance was considered as p≤0.05 (*p ≤ 0.05, **p ≤ 0.01, ***p ≤ 0.001) with respect to indicated groups (Mouse Image courtesy: Ms. Leena Sapra).

In the present study, we made an attempt to dissect the role of Bregs in modulating osteoclastogenesis. To determine whether Bregs possess the potential to modulate osteoclasts differentiation, we first induced Bregs under *in vitro* conditions. Bregs were thus generated as per established literature (9, 20). Firstly, B cells were purified from splenocytes of mice *via* negative selection through magnetic beads (**Figure 1A**). After assessing the purity of isolated B cells (>95%), we stimulated these purified B cells with LPS (10 ug/ml) for different time periods (5h and 24h). Since Bregs have already been reported to express IL-10, we too evaluated the frequency of CD19^+^IL-10^+^ Bregs in our study. Our flow cytometric data indicates that the frequency of total IL-10 producing B cells, i.e., CD19^+^IL-10^+^ Bregs was significantly enhanced by LPS stimulation in a time-dependent manner in comparison to the control group (p < 0.001) (**Figures 1B, C**). B10 Bregs with a characteristic phenotype of CD19^+^CD1d^hi^CD5^+^IL-10^+^ were also significantly enhanced after LPS stimulation with respect to control group (**Figure 1D**). In addition, our ELISA results also indicate that IL-10 cytokine levels were significantly enhanced in culture supernatant harvested from LPS stimulated Bregs in comparison to the control group (**Figure 1E**). These results thus clearly indicate towards the successful generation of IL-10 producing Bregs in our culture conditions.

### Bregs Inhibit Differentiation and Functional Activity of Osteoclasts

**Figure 2:**
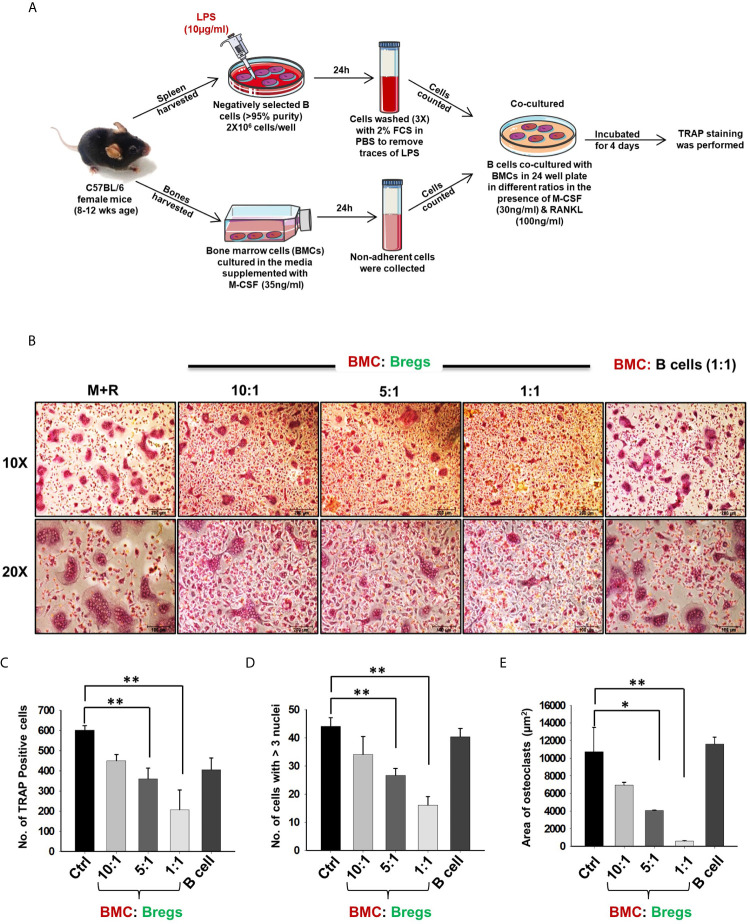
Bregs suppress osteoclastogenesis. **(A)** BMCs and LPS stimulated and non-stimulated B cells were co-cultured in cell culture plate (cell-contact) in the presence of M-CSF (30 ng/ml) and RANKL (100 ng/ml) for 4 days. B cells were induced with LPS (10 ug/ml) for 24h prior to co-cultures. **(B)** Bregs significantly inhibited the generation of multinucleated osteoclasts in a dose-dependent manner. **(C)** Graphical representation showing the number of TRAP-positive cells. **(D)** Bar graphs representing the number of cells with more than three nuclei. **(E)** Bar graphs representing the area of multinucleated TRAP-positive cells. Control denotes BMCs culture only. The above images are indicative of one experiment and similar results were obtained in three independent experiments. The results were evaluated by ANOVA with subsequent comparisons by Student t-test for paired or nonpaired data. Statistical significance was considered as p ≤ 0.05 (*p ≤ 0.05, **p≤0.01, ***p ≤ 0.001) with respect to indicated groups (Mouse Image courtesy: Ms. Leena Sapra).

Next, we wanted to investigate whether Bregs exhibit the potential to suppress osteoclast differentiation from BMCs under *in vitro* conditions. To confirm this, we co-cultured BMCs with Bregs at different cell ratios (BMC : Bregs:: 10:1, 5:1, and 1:1) in the presence of M-CSF (30 ng/ml) and RANKL (100 ng/ml). After incubation for 4 days, cells were fixed and stained for TRAP to identify differentiated multinucleated osteoclasts (**Figure 2A**). Interestingly, we observed that BMCs co-cultured with Bregs at different cell ratios showed a significant reduction in osteoclasts differentiation (3-fold), i.e., TRAP-positive osteoclasts (**Figures 2B–D**). Moreover, area measurement analysis of TRAP-positive multinucleated osteoclasts using Image J software also showed significant reduction (30-folds) in the area of osteoclasts in treated groups (**Figure 2E**). Thereby, our results clearly demonstrated that Bregs possess the potential to inhibit RANKL induced osteoclastogenesis in a dose-dependent manner.

**Figure 3:**
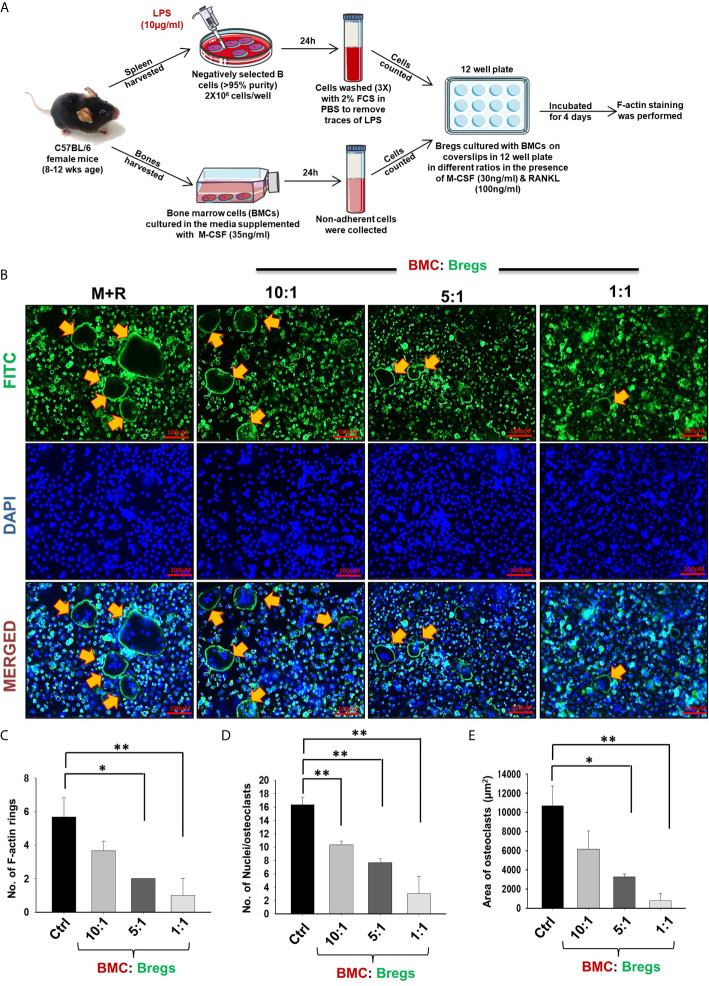
Bregs suppress F-actin formation in osteoclasts. **(A)** BMCs and Bregs were co-cultured in the presence of M-CSF (30 ng/ml) and RANKL (100 ng/ml) at different ratios for 4 days and at the end of incubation F-actin staining was performed. B cells were stimulated with LPS (10 ug/ml) for 24h prior to co-cultures. **(B)** F-actin and nuclei were stained with FITC conjugated phalloidin and DAPI respectively. Images were captured in a fluorescence microscope (Imager.Z2 Zeiss microscope) at 10 X magnification. **(C)** Number of F-actin rings **(D)** Number of nuclei per osteoclasts **(E)** Area of F-actin rings. The above images are indicative of one experiment and similar results were obtained in three independent experiments. The results were evaluated by ANOVA with subsequent comparisons by Student t-test for paired or nonpaired data. Statistical significance was considered as p≤0.05 (*p ≤ 0.05, **p ≤ 0.01, ***p ≤ 0.001) with respect to indicated groups (Mouse Image courtesy: Ms. Leena Sapra).

Extensive literature suggests that the F-actin ring observed in mature osteoclasts is a characteristic functional phenotype that aids toward the bone-resorbing potential of osteoclasts. Moving ahead, we next asked the question of whether Bregs have the potential to inhibit the functional activity of osteoclasts along with inhibiting their differentiation. In order to examine the same, Bregs and BMCs were co-cultured at different ratios on glass coverslips for 4 days. At the end of the experiment, after fixation and permeabilization, cells were stained for F-actin (FITC-labelled-phalloidin) and nuclei (DAPI). Remarkably, we observed that in comparison to the control group, Bregs significantly suppressed both the number of F-actin rings and the area of F-actin ring in matured osteoclasts (**Figures 3A–E**). These results of ours thus clearly establish the role of Bregs in significantly impairing not only the differentiation but also the functional capacity of osteoclasts.

### Bregs Inhibits Osteoclast Differentiation *via* IL-10

**Figure 4:**
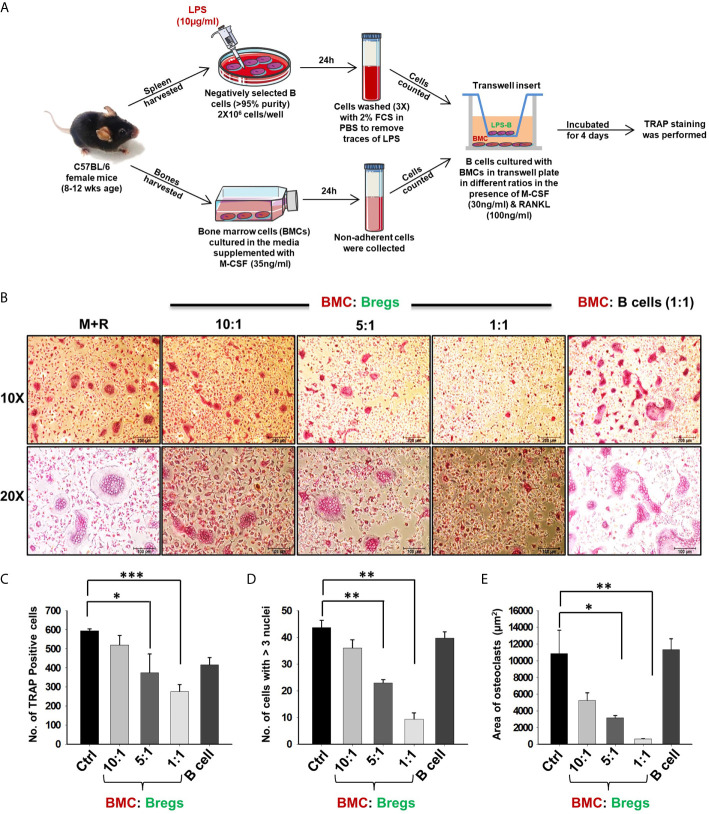
Bregs suppress osteoclastogenesis via soluble molecules. **(A)** BMCs and LPS stimulated and non-stimulated B cells were co-cultured in transwell insert in the presence of M-CSF (30 ng/ml) and RANKL (100 ng/ml) for 4 days. B cells were stimulated with LPS (10 ug/ml) for 24h prior to being added to co-cultures. **(B)** Bregs significantly inhibited the generation of multinucleated osteoclasts in a dose-dependent manner. **(C)** Graphical representation depicting the number of TRAP-positive cells. **(D)** Bar graphs representing the number of cells with more than three nuclei. **(E)** Bar graphs representing the area of multinucleated TRAP-positive cells. Control denotes BMCs culture only. The above images are indicative of one independent experiment and similar results were obtained in three independent experiments. The results were evaluated by ANOVA with subsequent comparisons by Student t-test for paired or nonpaired data. Statistical significance was considered as p≤0.05 (*p ≤ 0.05, **p ≤ 0.01, ***p ≤ 0.001) with respect to indicated groups (Mouse Image courtesy: Ms. Leena Sapra).

To dissect whether the suppressive effect of Bregs is mediated *via* either a cell-contact-dependent (soluble factors) or cell-contact-independent manner, we co-cultured BMCs and Bregs in trans-wells that prohibit direct cellular interactions. Remarkably, Bregs also significantly inhibited osteoclastogenesis in a dose-dependent manner (5-fold) even in a trans-well setup, thereby clearly establishing the role of soluble factors in mediating inhibitory anti-osteoclastogenic potential of Bregs (**Figures 4A–E**). These data clearly establish that Bregs inhibit osteoclast differentiation *via* soluble mediators.

**Figure 5:**
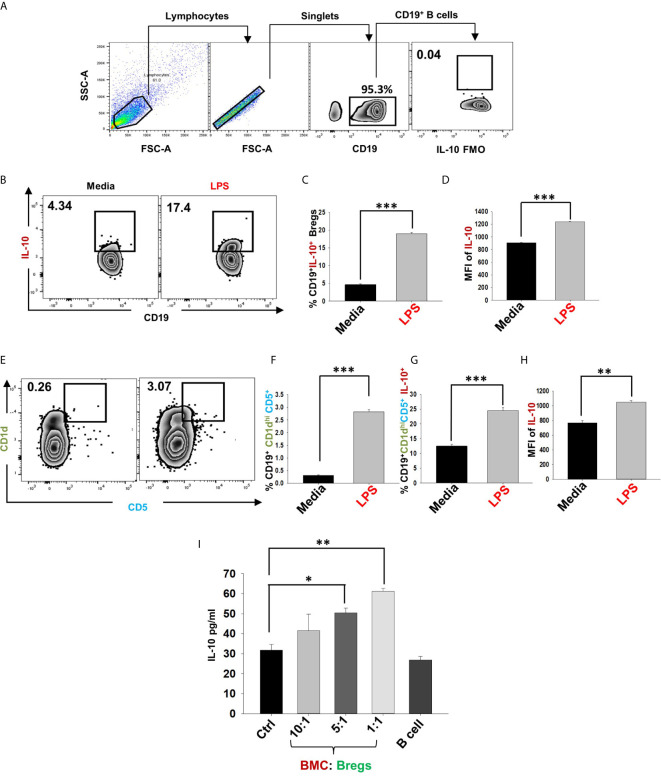
IL-10 producing Bregs inhibits osteoclastogenesis. After 24h of LPS stimulation cells were harvested and evaluated for estimating the expression of markers that may be associated with anti-osteoclastogenic function of B cells. **(A)** Gating strategy followed for data analysis. **(B)** Zebra plot depicting the percentages of CD19^+^IL-10^+^ Bregs in media and LPS induced Bregs. **(C)** Graphical representations depicting percentages of CD19^+^IL-10^+^ Bregs. **(D)** MFI of IL-10. **(E)** Zebra plot highlighting the percentages of CD19^+^CD1d^hi^CD5^+^ (B10) Bregs. **(F)** Graphical representation of CD19^+^CD1d^hi^CD5^+^ B10 Bregs. **(G)** Bar graph representing CD19^+^CD1d^hi^CD5^+^IL-10^+^ B10 Breg **(H)** MFI of IL-10 on CD19^+^CD1d^hi^CD5^+^. **(I)** ELISA results of supernatant harvested from co-cultures of BMCs and Bregs showing levels of IL-10 cytokine. The above images are indicative of one independent experiment and similar results were obtained in three independent experiments. The results were evaluated by ANOVA with subsequent comparisons by Student t-test for paired or nonpaired data. Statistical significance was considered as p≤0.05 (*p ≤ 0.05, **p ≤ 0.01, ***p ≤ 0.001) with respect to indicated groups.

Various studies have shown that LPS-induced Bregs mediate their immunosuppressive functions *via* IL-10 cytokine (20). Also, among various subtypes of Bregs, CD19^+^CD1d^hi^CD5^+^ (B10) Bregs are known to play a crucial role in preventing immunopathogenesis associated with various autoimmune diseases. Thus, it may be possible that the higher expression of IL-10 by these Bregs could contribute towards the inhibition of osteoclastogenesis in our co-cultures. Therefore, to confirm the significance of IL-10 in Bregs mediated suppression of osteoclastogenesis, we next assessed the percentages of total IL-10 producing B cells (CD19^+^IL-10^+^) along with B10 Bregs (CD19^+^CD1d^hi^CD5^+^IL-10^+^) in our LPS induced Bregs *via* flow cytometric analysis. Intriguingly, we observed that percentages of IL-10 producing Breg populations were significantly enhanced in the LPS induced group with respect to the control group (**Figures 5A–H**). Importantly, the anti-osteoclastogenic effect of Bregs was further found to be correlated with the significant enhancement in IL-10 cytokine levels in co-cultures supernatants in comparison to the control group (**Figure 5I**).

**Figure 6:**
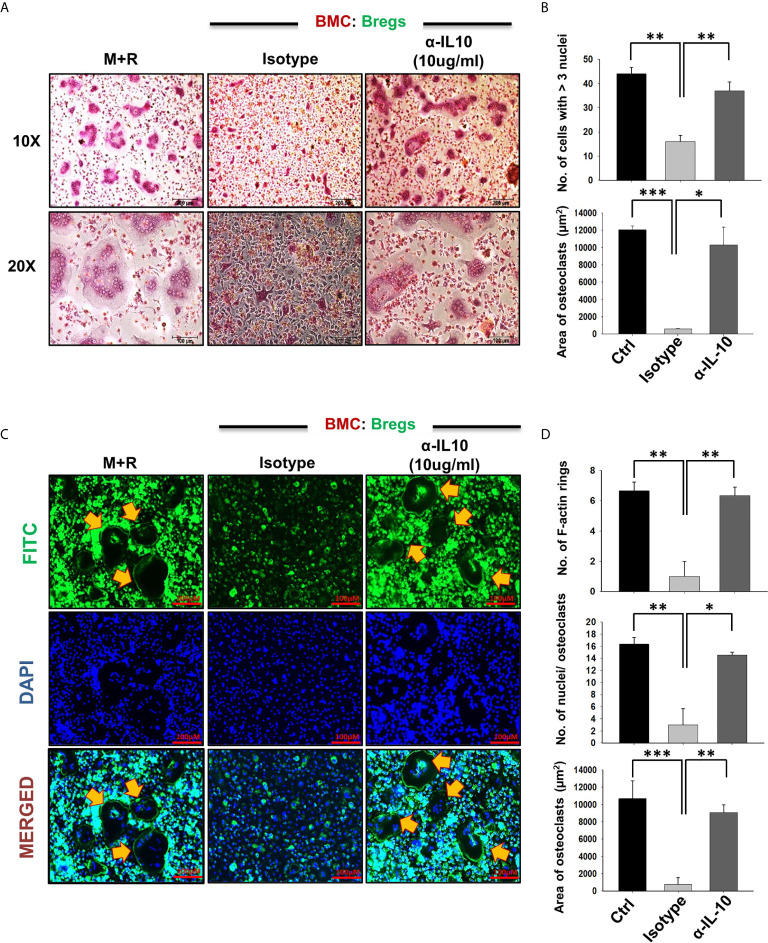
Bregs inhibit osteoclastogenesis in an IL-10 dependent manner. Osteoclastogenesis was evaluated after neutralization of IL-10 cytokine with antibodies against IL-10 in BMCs and Bregs co-cultures. **(A)** BMCs and LPS stimulated B cells were co-cultured (1:1) in 24 well plates in the presence of M-CSF (30 ng/ml) and RANKL (100 ng/ml) along with the presence or absence of α-IL10 & isotype control for 4 days. **(B)** Graphical representation of the number of cells with more than three nuclei and area of multinucleated TRAP-positive cells. **(C)** F-actin and nuclei were stained with FITC conjugated phalloidin and DAPI respectively. **(D)** Number of F-actin rings, Number of nuclei per osteoclasts, and Area of F-actin rings. The above images are indicative of one experiment and similar results were obtained in three independent experiments. The results were evaluated by ANOVA with subsequent comparisons by Student t-test for paired or nonpaired data. Statistical significance was considered as p≤0.05 (*p ≤ 0.05, **p ≤ 0.01, ***p ≤ 0.001) with respect to indicated groups.

To confirm whether the observed inhibitory effects of Bregs on osteoclastogenesis are mediated by IL-10 alone, we next performed an IL-10 neutralization assay. For the same, we employed anti-IL-10 MAb (10 ug/ml) to neutralize the anti-osteoclastogenic potential of Bregs in BMCs and Bregs cell co-cultures. Excitingly, we observed that anti-IL-10 significantly abolished the anti-osteoclastogenic potential of Bregs. Isotype control for anti-IL-10 was also set up to confirm these results (**Figures 6A–D**). Altogether, our results for the first time establish the direct role of Bregs in inhibiting *in vitro* osteoclastogenesis in an IL-10-dependent manner.

### Successful Development of Postmenopausal Osteoporotic Mice Model

**Figure 7:**
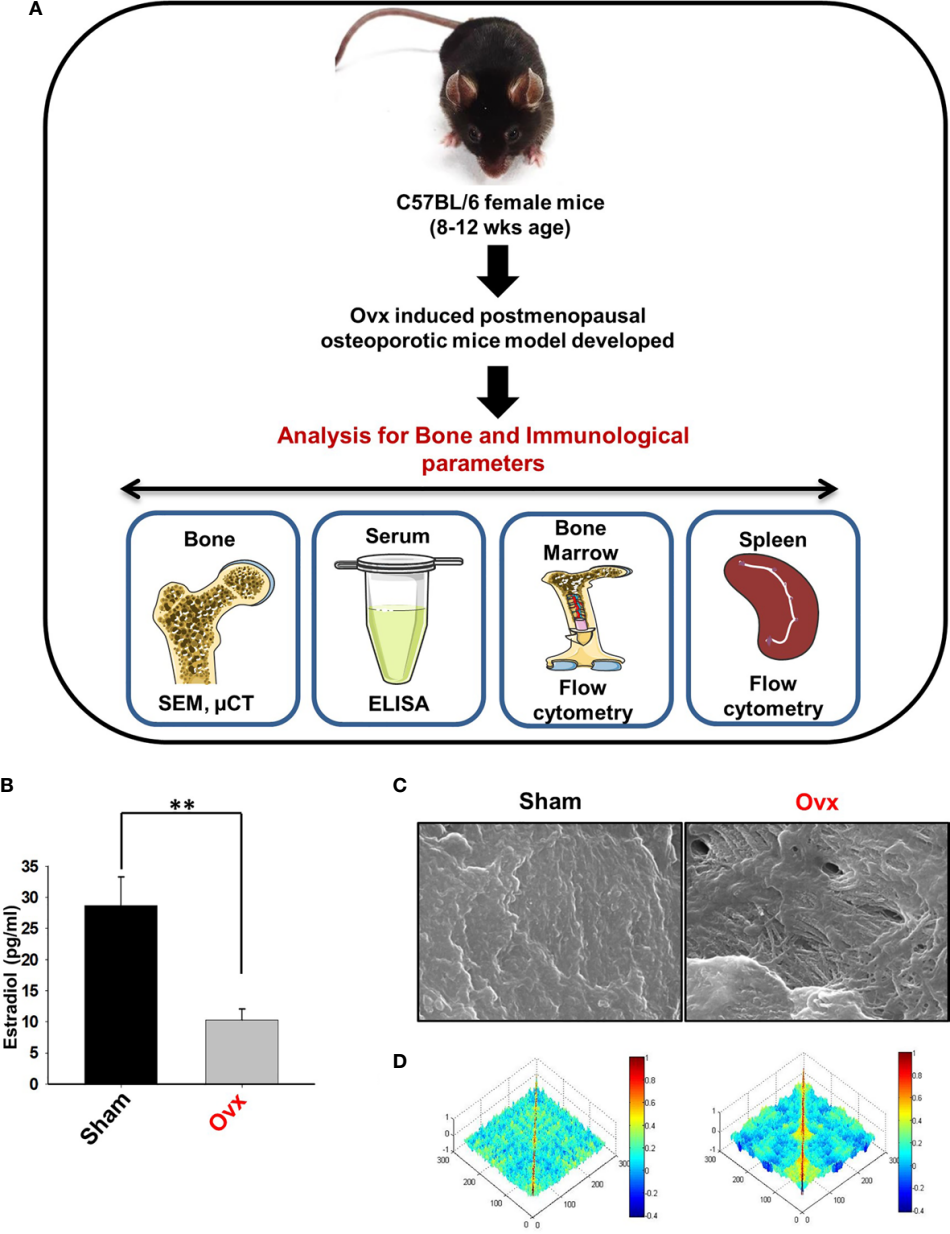
Development of postmenopausal osteoporotic mice model. **(A)** Experimental layout followed for *in vivo* study. **(B)** Estrogen level was estimated in blood serum of the sham and ovx mice groups. Mice were sacrificed at the end of the experiment and cortical bones of both the groups were collected for SEM analysis. **(C)** 2D SEM images. **(D)** MATLAB analysis of SEM images. The above images are indicative of one independent experiment and comparable results were obtained in two different independent experiments with n=6 mice/group/experiment. The results were evaluated by ANOVA with subsequent comparisons by Student t-test for paired or non-paired data. Values are reported as mean ± SEM. Similar results were obtained in two different independent experiments with n=6. Statistical significance was considered as p≤0.05 (*p ≤ 0.05, **p ≤ 0.01, ***p ≤ 0.001) with respect to indicated Sham group. (Mouse Image courtesy: Ms. Leena Sapra).

Next, to investigate the likely contribution of regulatory B cells in modulating bone health under normal and osteoporotic conditions, we firstly developed and authenticated a postmenopausal osteoporotic mice model, a prime requirement for our study. For accomplishing the same, female C57BL/6 mice were divided into two groups viz. sham and ovx. At the end of the experiment, blood serum was analyzed for estradiol levels, and bones were harvested for assessing the effect of estrogen deficiency on bone loss (**Figure 7A**). Our results clearly indicated a significant reduction in estradiol levels from 29 pg/ml (sham) to 9 pg/ml (3-fold) in the ovx group (p < 0.01) (**Figure 7B**).

After the successful development of a postmenopausal osteoporotic mice model, we next investigated the effect of estrogen loss on bone resorption and bone histomorphometric parameters. Scanning electron microscopy (SEM) and micro-computed tomography (µ-CT) analysis (a gold standard for determining bone health) were next performed. SEM analysis of cortical region (femoral bone) demonstrated an enhanced number of lacunae or resorption pits in the ovx group with respect to the control group (**Figure 7C**). To further analyze the SEM 2D-images in a statistical manner, we performed MATLAB (matrix-laboratory) analysis to determine the correlation between bone mass and bone loss in both groups. The MATLAB analysis specifies the degree of homogeneity where a red color indicates higher correlation values (high bone mass), and blue symbolizes lower correlation values (more bone loss). Outcomes of MATLAB analysis clearly showed that the ovx group has lesser correlation values or higher bone loss in the ovx group in comparison to the sham group (**Figure 7D**).

**Figure 8:**
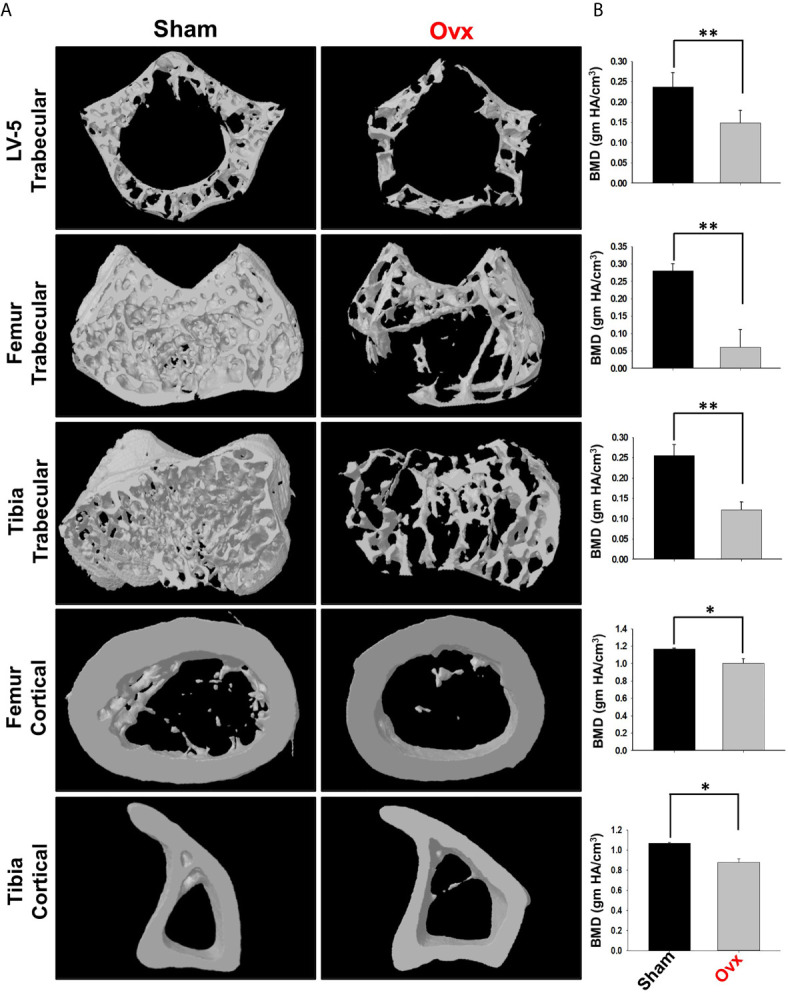
Micro-CT analysis of trabecular and cortical regions of Lumbar Vertebrae-5 (LV-5), Femoral and Tibial bones. **(A)** 3D micro-CT reconstructions of LV-5 trabecular region, Femur trabecular region, Tibia trabecular, Femur cortical, and Tibia cortical regions of the sham and ovx groups. **(B)** Graphical representations of Bone Mineral Density (BMD) of LV-5 trabecular region, Femur trabecular region, Tibia trabecular, Femur cortical, and Tibia cortical regions of the sham and ovx groups. The results were evaluated by ANOVA with subsequent comparisons by Student t-test for paired or non-paired data. Values are reported as mean ± SEM. Similar results were obtained in two different independent experiments with n=6. Statistical significance was considered as p≤0.05 (*p ≤ 0.05, **p ≤ 0.01, ***p ≤ 0.001) with respect to indicated Sham group.

Moving ahead, we next authenticated the successful development of osteoporotic conditions in our model *via* µ-CT analysis. As lumbar-vertebrae-5 (LV-5) is one of the peculiar regions to diagnose osteoporotic conditions (16). Therefore, we analyzed the effect of estrogen deficiency on the LV-5 trabecular region along with the trabecular region of femoral and tibial bones. Interestingly, µ-CT data revealed that estrogen deficiency significantly impaired the micro-architecture of LV-5, femoral, and tibial bones in the ovx group in comparison to the sham group (**Figure 8A**). Moreover, an ameliorated bone loss condition in the ovx group is further supported by significantly reduced bone mineral density (BMD) in the ovx group with respect to sham group (**Figure 8B**). The quantitative analysis of trabecular and cortical bones which provides an overview of the utmost fundamental histomorphometric indices that were derived from bone micro-architecture 3D images is shown in **Table 1**. Altogether, our results clearly established the successful development of the postmenopausal osteoporotic mice model.

**Table 1:** Bone Histomorphometric Parameters in Ovx mice.

Bone Parameters	Sham	Ovx
**LV5**		
BV/TV (%)	92.07 ± 2.84	60.87 ±6.62**
Tb. Th (mm)	0.06 ± 0.001	0.04 ± 0.001**
Tb. No (mm^-1^)	11.8 ± 0.86	2.49 ± 0.08**
Conn. Den (mm^-3^)	183.05 ± 43	89.66 ± 6.1
Tb. Sp. (mm)	0.01 ± 0.00	0.27 ± 0.003***
Tb. Pf (mm^-1^)	18.4 ± 0.22	29.7 ± 0.64*
**Femur Trabecular**		
BV/TV (%)	60.93 ± 3.21	8.24 ±1.18**
Tb. Th (mm)	0.068 ± 0.005	0.04± 0.003*
Tb. No (mm^-1^)	13.6 ± 0.8	1.44 ± 0.18**
Conn. Den (mm^-3^)	227 ± 29.6	58.4 ± 6.02**
Tb. Sp. (mm)	0.07 ± 0.01	0.66 ± 0.03**
Tb. Pf (mm^-1^)	11.12± 1.54	26.11 ± 0.80**
**Tibia Trabecular**		
BV/TV (%)	88.93 ± 0.09	5.23 ± 0.15***
Tb. Th (mm)	0.07 ± 0.001	0.05 ± 0.0006**
Tb. No (mm^-1^)	13.1 ± 0.45	1.1 ± 0.15***
Conn. Den (mm^-3^)	60.24 ± 3.88	24.79 ± 0.95**
Tb. Sp. (mm)	0.04 ± 0.002	0.53 ± 0.01***
Tb. Pf (mm^-1^)	15.14 ± 1.01	26.43 ± 0.78**
**Femur Cortical**		
Tt. Ar (mm^2^)	2.22 ± 0.05	1.5 ± 0.19*
T. Pm (mm)	5.69 ± 0.09	4.95 ± 0.06*
Ct. Ar (mm^2^)	0.85 ± 0.007	0.63 ± 0.04*
B. Pm (mm)	10.33 ± 0.17	8.95 ± 0.15*
Ct. Th (mm)	0.17 ± 0.001	0.12 ± 0.01*
**Tibia Cortical**		
Tt. Ar (mm^2^)	1.46 ± 0.012	1.23 ± 0.02**
T. Pm (mm)	7.4 ± 0.79	5.7 ± 0.25
Ct. Ar (mm^2^)	0.70 ± 0.012	0.58 ± 0.02*
B. Pm (mm)	10.4 ± 0.2	9.06 ± 0.25*
Ct. Th (mm)	0.15 ± 0.001	0.11 ± 0.004**

BV/TV, Bone volume/tissue volume ratio; Tb. Th, trabecular thickness; Tb. No., trabecular number; Conn. Den, connectivity density; Tb. Sep., trabecular separation; Tb. Pf., trabecularpattern factor; Tt. Ar., total cross-sectional area; T. Pm, total cross-sectional perimeter; Ct. Ar, cortical bone area; B. Pm, bone perimeter; Ct. Th, average cortical thickness. The results were evaluated by ANOVA with subsequent comparisons by Student t-test for paired or nonpaired data. Values are reported as mean ± SEM. Similar results were obtained in two different independent experiments with n=6. Statistical significance was considered as p≤0.05 (*p ≤ 0.05, **p ≤ 0.01, ***p ≤ 0.001) with respect to indicated Sham group. Histomorphometric indices of LV-5 trabecular, femur trabecular, tibia trabecular, femur cortical, and tibia cortical of the sham and ovx mice groups.

### Bregs Play an Important Role in Ameliorating Ovariectomy-Induced Bone Loss

**Figure 9:**
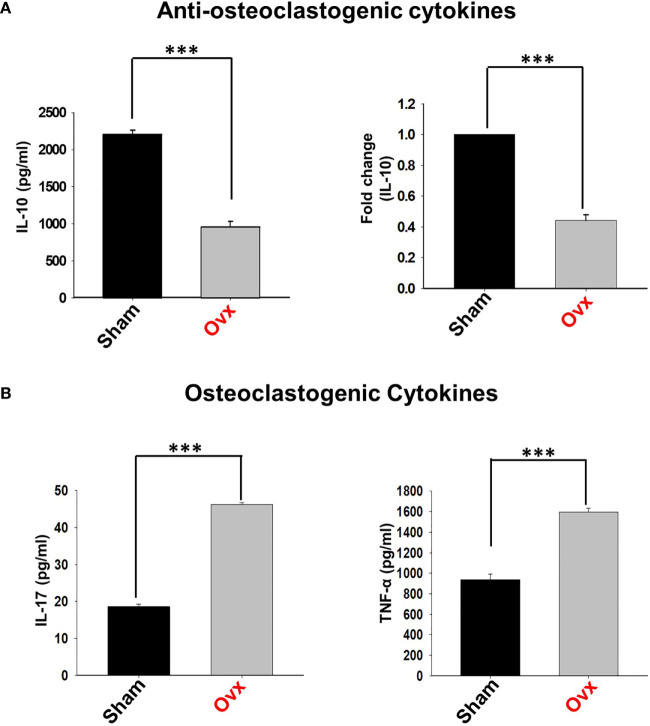
Osteoclastogenic and anti-osteoclastogenic cytokine levels in ovx mice. Cytokines were analyzed in serum samples of mice by ELISA. **(A)** Levels of IL-10 cytokine. **(B)** Levels of IL-17 and TNF-α cytokines in the sham and ovx groups. The results were evaluated by using ANOVA with subsequent comparisons by Student t-test for paired or non-paired data, as appropriate. Values are expressed as mean ± SEM (n=6) and similar results were obtained in two independent experiments. Statistical significance was defined as p≤0.05, *p ≤ 0.05, **p < 0.01 ***p≤0.001 with respect to indicated mice group.

**Figure 10:**
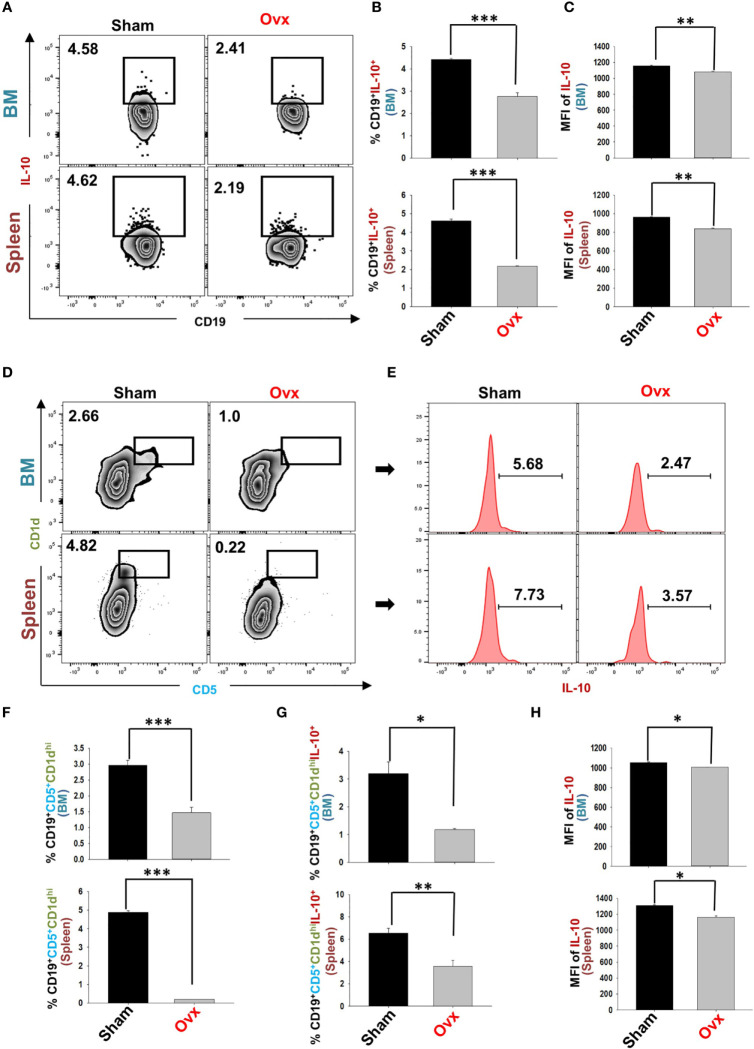
Ovariectomy results in modulation of Bregs population in vivo. Cells from Bone Marrow (BM) and spleen of the sham and ovx groups were harvested and analyzed by flow cytometry for the percentage of Breg **(A)** Zebra plot depicting percentages of CD19^+^IL-10^+^ B cells in BM and spleen of sham and ovx. **(B)** Bar graph representing percentages CD19^+^IL-10^+^ Bregs in sham and ovx. **(C)** Bar graphs representing the MFI of IL-10 in BM and spleen. **(D)** Zebra plot depicting percentages of CD19^+^CD1d^hi^CD5^+^ B10 cells in BM and spleen. **(E)** Histograms representing IL-10 cytokine levels in the sham and ovx groups. **(F)** Graphical representation of CD19^+^CD1d^hi^CD5^+^ Bregs in sham and ovx mice. **(G)** Graphical representation of the percentage of CD19^+^CD1d^hi^CD5^+^IL-10^+^ B10 cells in sham and ovx mice. **(H)** Graphical representation of MFI of IL-10 in sham and ovx. The results were evaluated by using ANOVA with subsequent comparisons by Student t-test for paired or non-paired data, as appropriate. Values are expressed as mean ± SEM (n=6) and similar results were obtained in two independent experiments. Statistical significance was defined as p≤0.05, *p ≤ 0.05, **p < 0.01 ***p ≤ 0.001 with respect to indicated mice group.

Estrogen-deficiency-induced bone loss is mediated by various factors viz. immunological, biochemical, etc. Also, the role of cytokine imbalance in regulating bone health is well established. Results from our lab (13, 14) along with others have reported significantly reduced levels of osteoprotective cytokine IL-10 along with higher levels of osteoclastogenic cytokines viz. IL-17 and TNF-α under ovx conditions (**Figures 9A, B**). Extensive literature suggests that the major source of IL-10 is immune cells. Recently, several studies have reported that apart from Tregs, Bregs are also a major source of IL-10 under physiological conditions (6, 21). Therefore, a strong possibility exists that the observed reduction of IL-10 levels under osteoporotic conditions may not be primarily due to dysregulation of Tregs alone. Based on these studies we strongly believe and propose that alteration in the Bregs population could also be one of the major contributing factors for observed significant reduction in IL-10 levels observed under ovx conditions. Thus, we next asked the question: are the observed significantly reduced levels of IL-10 in ovx mice due to dysregulation of the Bregs population? We thus analyzed the frequency of Bregs in both the BM (prime site of osteoclastogenesis) and spleen (main site of Bregs induction) in mice. Interestingly, the frequencies of total IL-10 producing B cells, i.e., CD19^+^IL-10^+^ cells were significantly reduced in the ovx group in comparison to the control group (p < 0.01) in both BM and spleen (**Figures 10A–C**). Notably, the population of “B10” Bregs, i.e., CD19^+^CD1d^hi^CD5^+^IL-10^+^ were also observed to be significantly reduced in both BM and spleen of the ovx group in comparison to th sham group (p < 0.05) (**Figures 10D–H**). Taken together, our results clearly point towards a crucial role of Bregs in modulating bone loss under postmenopausal osteoporotic conditions.

## Discussion

Studies from our lab along with others have highlighted the relevance of the immune system in maintaining bone health under both physiological and osteoporotic conditions (13, 19, 22, 23). These studies laid the foundation for the importance of the immune system in osteoporosis, i.e., “Immunoporosis” (19). Various studies demonstrated that the homeostatic balance between anti-inflammatory (Tregs) and inflammatory (Th17) cells is of utmost importance in various diseases including osteoporosis (13, 16, 24). Apart from Tregs, there are also other regulatory lymphocytes with established immunosuppressive functions. Most important among these are the recently discovered “Bregs”.

Bregs with immunoregulatory potential have been observed in several disease models of autoimmunity, such as RA, SLE, experimental autoimmune hepatitis (EAH), etc. (6, 21, 25). Bregs perform their regulatory functions *via* secreting soluble cytokines (viz. IL-10) or expressing membrane-bound surface molecules (TGF-β). Nevertheless, the primary regulatory mechanism of Bregs is based on the immunoregulatory cytokine IL-10. Multiple studies demonstrated that IL-10 is an anti-osteoclastogenic cytokine that maintains bone health by inhibiting osteoclastogenesis (26, 27). Moreover, the significance of IL-10 is further highlighted in IL-10 deficient mice in which all the hallmarks of osteoporosis i.e. decreased bone mass, enhanced mechanical fragility, and reduced bone formation was observed (28). Moreover, Tregs *via* secreting IL-10 and other cytokines viz. IL-4 along with membrane-bound receptors, such as cytotoxic T lymphocyte antigen (CTLA)-4 abrogates osteoclastogenesis (15). IL-10 is a pivotal immunomodulator in infectious diseases, autoimmune disorders, inflammatory bone loss conditions including osteoporosis, but no study to date has ever investigated the significance of IL-10 secreting Bregs in regulating bone health in both *in vitro* and *in vivo* conditions. Multiple studies suggested that Bregs mediate its immunosuppressive functions by promoting the differentiation of naïve CD4^+^ T cells into Tregs (29, 30). Importantly, a study also suggested that even in the absence of Tregs, Bregs suppressed arthritis thereby highlighting towards the independent function of Bregs (31).

IL-10 is an established regulatory molecule of Bregs; but the expression of IL-10 cytokine is quite low in resting B cells, thus to enhance the expression of IL-10, *in vitro* stimulation of B cells is required. A study reported by Yang et al. (32), demonstrated that LPS stimulation for 24h leads to enhancement of IL-10 producing Bregs (32). Thus, we chose this particular method for the generation of Bregs in our experiments. Next, we activated purified B cells (>95%) with LPS for different time periods (5h and 24h) and, in consistence with previously reported studies, we too observed a time-dependent induction of IL-10 producing Bregs. Moving ahead in our study, we were further interested in investigating the role of Bregs in modulating RANKL induced osteoclastogenesis under *in vitro* conditions. Excitingly, we observed that Bregs inhibited the differentiation of osteoclasts from BMCs in a dose-dependent manner. Also, our *in vitro* data suggested that Bregs exhibit the potential of suppressing F-actin ring formation in osteoclasts: a vital characteristic phenotype of mature osteoclasts responsible for their functional activity (bone resorption). We were further keen to know whether the observed suppression of osteoclastogenesis by Bregs is cytokine dependent (soluble factors) or requires cell-to-cell contact. Thus, we employed a trans-well system for our cultures and observed that the anti-osteoclastogenic potential of Bregs is primarily mediated by soluble factors. In addition to this, both our ELISA results (co-culture supernatant) along with flow cytometric data of cells harvested from the co-cultures of BMCs and Bregs clearly establish the role of IL-10 secreting Bregs in suppressing osteoclastogenesis. Moreover, our anti-IL-10 neutralizing antibody experiment clearly showed restoration of osteoclastogenesis in BMCs even in the presence of Bregs, thereby establishing the role of IL-10 in Bregs mediated regulation of osteoclastogenesis. Altogether, these findings of ours for the first time establish the direct role of Bregs in inhibiting osteoclastogenesis under *in vitro* conditions.

A study reported in 2013 by the Flores et al. group demonstrated that numerical defects or profound reduction in frequencies of Bregs as a major causative factor for RA in humans (6). In the beginning of 2020, our group also highlighted the probable contribution of IL-10 producing regulatory B cells in the case of osteoporosis and found that IL-10 producing Bregs are significantly reduced in the post-menopausal osteoporotic mice model (unpublished data) (1). As is consistent with our data, recently, one study also suggested that Bregs exhibit osteoprotective potential in case of post-menopausal osteoporosis as adoptive transfer of IL-10 producing Bregs into ovx-mice-reduced bone deterioration (33). In line with these findings, our present study, reports the modulation of IL-10 secreting Bregs not only in the spleen but also in BM, the prime site of osteoclastogenesis. Furthermore, our *in vivo* flow cytometric data clearly indicates a numerical defect in both CD19^+^IL-10^+^ Bregs and CD19^+^CD1d^hi^CD5^+^IL-10^+^ “B10” cells in both BM and spleen (main site of Bregs generation). Furthermore, our serum cytokine data corroborate our flow cytometric data with significant reduction of IL-10 levels in osteoporotic group with respect to control group.

**Figure 11:**
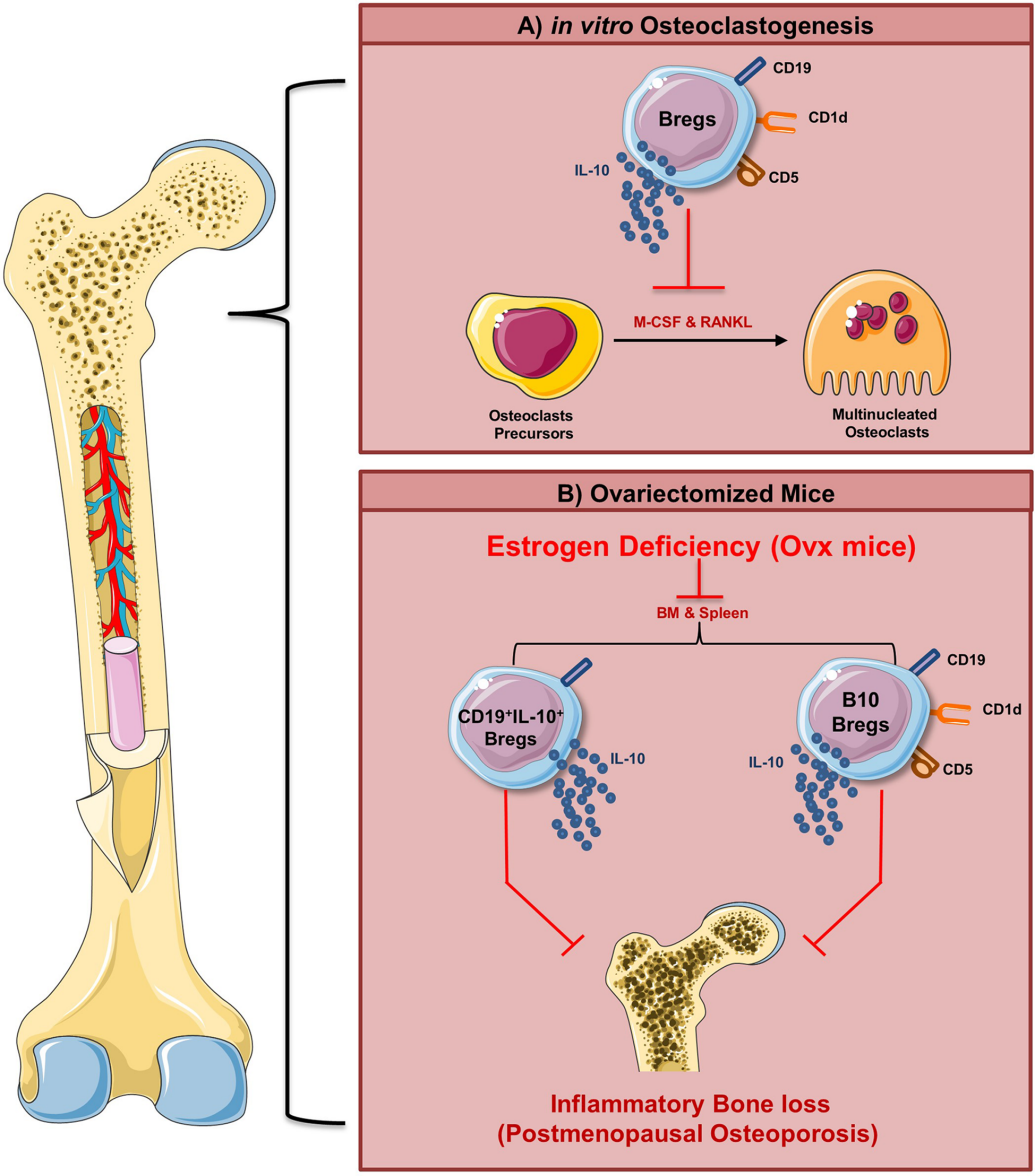
Summary of results. **(A)** Regulatory B cells inhibit RANKL induced osteoclastogenesis under *in vitro* conditions via IL-10 cytokine. **(B)** Our *in vivo* data suggest that modulation of the Bregs population (both bone marrow and spleen) along with its reduced potential to produce IL-10 ameliorates inflammatory bone loss observed under postmenopausal conditions in ovx mice (Image illustrated using Medical Art https://smart.servier.com/).

Taken together, our present study for the first time establishes that Bregs exhibits anti-osteoclastogenic potential *in vitro*. Moreover, reduction in Bregs number observed *in vivo* may also be one of the prime contributing factors towards inflammatory bone loss observed in the postmenopausal osteoporotic mice model. These results thus provide novel insight into Bregs biology in the context of osteoporosis (**Figure 11**). Moreover, apart from these Bregs populations (viz. CD19^+^IL-10^+^ and CD19^+^CD1d^hi^CD5^+^IL-10^+^ Bregs), we also observed that other Bregs populations with characteristic phenotypes of CD19^+^FOXP3^+^ Bregs and CD19^+^CD11b^+^ Bregs were also found to be decreased in the case of an osteoporotic mice model (unpublished observation). Thus, further studies are need of the hour to fully dissect and establish the role of various populations of Bregs in post-menopausal osteoporotic conditions, which would thereby lead to future employment of Bregs-based cellular therapy in ameliorating inflammatory bone loss observed in osteoporosis.

## Data Availability Statement

The raw data supporting the conclusions of this article will be made available by the authors, without undue reservation.

## Ethics Statement

The animal study was reviewed and approved by Institutional Animal Ethics Committee of AIIMS, New Delhi, India (85/IAEC-1/2018 and 196/IAEC-1/2019).

## Author Contributions

RS contributed to the conceptualization and investigation of the study. LS and AB contributed to the methodology and formal analysis of data. PM carried out cytokine analysis. RS and LS contributed to the writing and editing of the manuscript. GM, BG, and BV provided valuable inputs in the study design. All authors reviewed the manuscript. All authors contributed to the article and approved the submitted version.

## Funding

This work was financially supported by projects: DST-SERB (EMR/2016/007158), Govt. of India and intramural project from All India Institute of Medical Sciences (AIIMS, A-798), New Delhi-India sanctioned to RS; National Academy of Sciences (NASI), Allahabad-India sanctioned to GM.

## Conflict of Interest

The authors declare that the research was conducted in the absence of any commercial or financial relationships that could be construed as a potential conflict of interest.

## Abbreviations

Bregs, Regulatory B cells; RANKL, Receptor activator of nuclear factor kappa B; TLR, Toll like receptor; LPS, Lipopolysaccharide; IL, Interleukin; TGF, Transforming growth factor; RA, Rheumatoid arthritis; SLE, Systemic lupus erythematosus; EAE, Experimental autoimmune encephalitis; PMA, Phorbol 12-myristate 13-acetate; BMCs, Bone marrow cells; M-CSF, Monocyte colony stimulating factor; TRAP, Tartrate resistant acid phosphatase; BMD, Bone mineral density; SEM, Scanning electron microscopy; Tregs, Regulatory T cells CIA; Collagen induced arthritis.
